# Clinical practice guideline of the Spanish society of oral surgery for dental care in patients with oral cancer

**DOI:** 10.4317/medoral.26064

**Published:** 2023-06-18

**Authors:** María Baus-Domínguez, María Rizo-Gorrita, Cristina Herráez-Galindo, Sara Bakkali, Íñigo Fernández-Figares-Conde, Mª Ángeles Serrera-Figallo, Aida Gutiérrez-Corrales, Celia Vázquez-Pachón, Javier Alberdi-Navarro, Agurne Uribarri-Etxebarría, Leticia Bagán-Debón, MªÀngels Sánchez-Garcés, José Luis Gutiérrez-Pérez, Daniel Torres-Lagares

**Affiliations:** 1 Department of Oral Surgery, Faculty of Dentistry, University of Seville, Seville, Spain; 2 Department of Stomatology, Faculty of Medicine and Nursing, University of País Vasco, Bilbao, Spain; 3 Department of Stomatology, Faculty of Medicine and Dentistry, University of Valencia, Valencia, Spain; 4 Department of Stomatology, Faculty of Medicine and Health Sciences, University of Barcelona, Barcelona, Spain; 5 Oral and Maxillofacial Unit, Virgen del Rocio Hospital, Seville, Spain

## Abstract

**Background:**

Oral cancer is the sixteenth most common malignant neoplasm worldwide, with a high mortality rate, greater than 50% at five years, and high morbidity. The effect of oncological treatment in the oral cavity is broad and has multiple levels, therefore knowing these effects and preventing them is essential for avoiding an increase in the oral pathology related with oncological therapy, maintaining the quality of life of the patient, and improving the efficacy of the treatment itself.

**Material and Methods:**

A group of experts belonging to the fields of Dentistry, Maxillofacial Surgery and Oncology of the University of Seville and the Virgen del Rocío University Hospital of Seville in collaboration with the University of Valencia, University of Barcelona, and University of the Basque Country, developed this Clinical Practice Guideline for the proper clinical management of patients diagnosed with oral cancer. The clinical questions were formulated in PICO format. The databases consulted were Medline/PubMed and Embase/Elsevier. The systematic reviews published on the topic were identified on Tripdatabase, Cochrane Library and CRD (Centre for Reviews and Dissemination). The recommendations were prepared based on the GRADE methodology.

**Results:**

Various recommendations were defined, derived from the 21 PICO questions, referring to prevention, treatment and care for alterations arising from the pathology of oral cancer itself and its treatment.

**Conclusions:**

The preparation of this clinical practice guideline allows recommendations to be generated based on the scientific evidence available, on dentistry actions in patients with oral cancer and undergoing oncological treatment, which may be of use to the multidisciplinary team treating this type of patient.

** Key words:**Oral cancer, oncology, clinical practice guideline, oral surgery.

## Introduction

Today, head and neck cancer is the seventh most common cancer worldwide. Each year, 660,000 new cases (1-3) and around 325,000 deaths (4,5) are recorded. According to 2020 data from the Global Cancer Observatory (GLOBOCAN), oral cancer is the 16th most common neoplasia, with over 350,000 cases annually.

Approximately 90-95% of oral cancers are squamous cell carcinoma (SCC). Its aetiology is multifactorial and has historically been associated with tobacco consumption, among other factors, being the most significant risk factor for developing oral cancer (6-10). However, potential changes in its etiopathogenesis are proposed given the reduction of tobacco use, particularly in developed countries (11).

The number of oral cancers has increased considerably in the elderly population, largely due to increased longevity. It is therefore estimated that in the next 20 years there will be a global increase of 66.2% in the number of new cases in this population (7).

Despite the numerous scientific-technological advances in terms of oral cancer treatment, it continues to have a high mortality rate, with over 150,000 deaths per year according to the 2020 data from GLOBOCAN. Its treatment therefore continues to be a great challenge, as survival rates have not significantly improved in recent decades (6,7). All of this highlights the importance of undertaking correct prevention and adequate early diagnosis.

In broad terms, oral cancer can be treated by surgery, chemotherapy and radiation therapy, administered independently or in combination.

The effect of the oncological treatment on the oral cavity is broad and may have multiple levels. Knowing the side effects and their severity, being able to diagnose, treat and prevent them is essential for avoiding an increase of the oral pathology, preserving the quality of life of the patient, and improving the efficacy of the oncological treatment itself. It is therefore imperative to evaluate the best evidence available to offer the dental care to the patient with oral cancer, as well as organising and offering it in an appropriate way to serve both the professional and the patient, to thereby be able to work under recommendations based on evidence of the highest possible quality.

Although there are precedents in Clinical Practice Guidelines in the field of Oral Cancer, none focus on the most specific aspects of the role of the Dentist. The aspects which led to the creation of this clinical guideline focused on the action of the dentist lie in the existence of groups with proven experience within the Spanish Society of Oral Surgery for approaching this project with sufficient guarantees, as well as it being an area of clinical importance, because there are unfortunately many patients facing the challenge of overcoming oral cancer.

## Material and Methods

- Development of the cooperative proposal

A Clinical Practice Guideline is defined as a “systematised set of recommendations which has the objective of helping the healthcare professional and the patient to adopt the most suiTable measures against a specific health issue”.

The cooperative proposal for the development of a clinical practice guideline for “Dental Care for Patients with Oral Cancer” began at the start of 2019 by the Spanish Society for Oral Surgery, due to the non-existence of a protocol based on scientific evidence which set out, in one document, all aspects to take into account and recommendations when providing dental care to patients who suffer from or have suffered from oral cancer.

- General approach

The Spanish Society for Oral Surgery, as a scientific association, has expertise in research and continuous training of its members. The Preparation of the Clinical Practice Guideline for Dental Care for Patients with Oral Cancer was completed on the 21st of June 2021 and was approved for publication on the 1st of March 2023 in the GuíaSalud Catalogue of the body of the National Health System (SNS) in which the 17 Autonomous Regions and the Ministry of Health of Spain participate, with the objective of improving the quality of healthcare in the SNS (https://portal.guiasalud.es/gpc/atencion-odontologica-al-paciente-con-cancer-oral/).

The guideline is designed with the objective of improving diagnosis and providing recommendations on dental care in any of the phases of the process of care for the cancer patient. It is therefore divided into three sections: Dental therapeutic approach for the patient with cancer from diagnosis to the start of the oncological treatment; Dental therapy provided to the patient during their oncological treatment; Dental care for the patient after completion of their oncological treatment.

- Constitution of the panel of experts

The panel of experts was made up of a multidisciplinary team of oral surgeons, maxillofacial surgeons, oncologists and dentists specialised in oral medicine and dentistry for patients with special needs, from the Faculty of Dentistry of the University of Seville and the Virgen del Rocío University Hospital, in collaboration with the University of Valencia, University of Barcelona and University of the Basque Country.

All participants worked under the guidelines of technical experts in Methodology and Healthcare based on Evidence. One of them was specialised in methodology of clinical practice guidelines and care processes; while the other technician was a librarian specialised in the field of health with a Diploma in Epidemiology and Research.

A coordinator was designated, in charge of resolving clinical doubts, as well as providing terminology for the search in free language and approving different search strategies for each PICO question.

The coordinators of the PICO questions were responsible for filtering manuscripts which were of interest for preparing the answers to the questions. All articles were classified in different folders named by labels for consultation by the panel of experts.

All members of the panel of experts completed the conflict of interests form, with no member having any conflict of interest which would impede their participation.

- Preparation of clinical questions and PICO questions. Schematisation of answers.

The panel of experts that has prepared this clinical practice guideline was governed by the indications of the document “Preparation of Clinical Practice Guidelines of the National Health System. Methodological Manual” of the working group for updating the Manual for the Preparation of Clinical Practice Guidelines in the Spanish National Health System, and the recommendations available on GuíaSalud (https://portal.guiasalud.es/wp-content/uploads/2019/01/manual_gpc_completo.pdf).

For each clinical question, a work sheet was created in which the following aspects were detailed:

1. issue raised: clinical questions seeking specific answers applicable in daily clinical practice for specific health problems, and which arise from expert knowledge in the field and the experience of each professional.

2. PICO Question (Patient/Population/Problem; Intervention/exposure; Comparison/Control; Outcome). The definition of the question and undertaking of the bibliographic search were thereby improved.

3. Introduction: Summary of knowledge based on the scientific evidence published in relation with the pathology and/or doubt raised, accompanied by its PICO question and recommendation.

4.Type of question: epidemiological/etiological, diagnostic, therapeutic or prognostic.

5. Methodology used: EUnetHTA “Process of information retrieval for systematic reviews and health technology assessments on clinical effectiveness” developed by the German Institute for Quality and Efficiency in Health Care (IQWiG).

a. Databases: PubMed/MEDLINE, Embase/Elsevier, Tripdatabase, Cochrane Library and Centre for Reviews and Dissemination (CRD). The search was started on Embase as it provides both unique content and complete content of MEDLINE. Additionally, the number of terms on Emtree (Embase thesaurus) is three times larger than MeSH (MEDLINE thesaurus).

b. Design of the search strategy using Boolean operators (AND, OR, NOT) along with the MeSH or Emtree keywords or terms. The research question was divided into concepts, and only the most important were used to develop the search strategy (generally population, intervention and study type). The English, French and Spanish languages were established as filters.

c. Results obtained: all bibliographic references resulting from the search were shared in real time, as with strategies and reference on Google Drive and using the flow diagram of PRISMA 2009 (12).

6. Evaluation and synthesis of the evidence: This section constitutes the evaluation of the quality of scientific evidence on which the recommendations of the guide are based. Critical reading of the references obtained in the search was carried out through the “critical appraisal tools”.

For the evaluation of randomised controlled clinical trials (RCTs), systematic reviews and meta-analysis, cohort studies, case control studies, diagnostic test studies and economic evaluations, the Scottish Intercollegiate Guidelines Network (SIGN) Methodology Checklist was used (13).

For the evaluation of case series, OSTEBA was used (Healthcare Technologies Evaluation Service of the Ministry of Health, Social Services and Equality of the Government of Spain), available on https://www.ser.es/wp-content/uploads/2018/04/Informe-OSTEBA.-FLC-3.0.pdf.

For the evaluation of clinical practice guidelines, the Appraisal of Guidelines Research and Evaluation (AGREE) was used (14).

7. Conclusions: recommendations are included on the PICO raised, as well as grading of this recommendation following the GRADE methodology (Grading of Recommendations, Assessment, Development, and Evaluations) which allows the evaluation of the quality of evidence and grading of the “strength” of the recommendations (15), corresponding with the letters A, B, C or D and whose meaning is set out in Table 1.

8. Recommendations for future research: during the preparation of a clinical practice guideline where an exhaustive review of the literature is carried out, questions not previously raised and which lack answers may arise, therefore being of interest for future research.

- Chronogram

A general overview of the dental management of the cancer patient was possible thanks to an initial bibliographic search through the PubMed and Embase databases to ascertain the current situation of the patient with oral cancer at the dental consultation. They key questions and the identification of existing systematic reviews and potentially relevant primary studies were necessary for the overall preparation of the project.

This first bibliographic search carried out in the first half of 2019 was used to define the scope of the clinical practice guide that was intended to be prepared. The magnitude of the issue and the variability in daily clinical practice, in addition to the high costs of care for this type of patient, were the bases used to design this clinical practice guideline, whose draft was presented in October 2019. From then, the criteria for beginning preparation of the PICO questions were defined, which ended by December 2020.

**Table 1:**
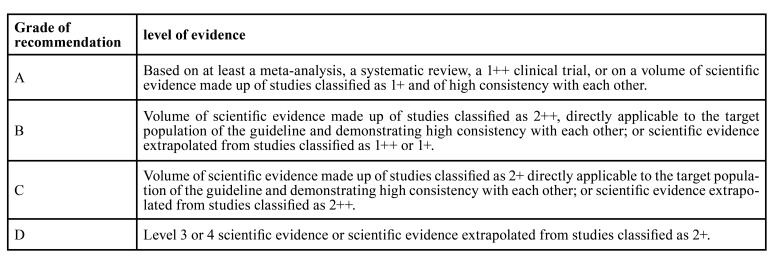
Summary of evidence and recommendations on clinical practice according to GRADE (Grading of Recommendations, Assessment, Development, and Evaluations).

Between January and July 2020, the bibliographic searches were carried out, through designed search strategies that you can consult in ANNEX 1 of the Clinical Practice Guideline at the following link https://portal.guiasalud.es/wp-content/uploads/gravity_forms/2-cdb008c5a6f7016ab2b9afa9675d272f/2023/02/Cancer-FINAL.pdf and peer reviewed using the PRESS 2015 Guideline Evidence-Based Checklist (16).

All references and full texts were downloaded on Mendeley and, thanks to review by the panel of experts and weekly alerts, it was possible to detect manuscripts of interest which would have been omitted from the searches and to update the material used. Between August and October of that year, some of the PICO questions were redefined, which due to their structure could not be answered, and the information was completed to answer these questions with new bibliographic searches carried out from October to the start of December 2020.

By then, part of the panel of experts had already completed the datasheet designed for each PICO question. With all the information, based on the scientific evidence available, the recommendations and grade of evidence for each one were drafted.

From January to April 2021, the Clinical Practice Guide was written, presented for distribution in June 2021. As previously stated, it was not until the 1st of March that the guide was evaluated with the six inclusion criteria of the GuíaSalud Catalogue, fulfilling all of them satisfactorily and being accepted for inclusion.

## Results

- Summary of recommendations before beginning the oncological treatment

Clinical Question 1: Are tobacco use and alcohol abuse related with a higher rate of oral complications in adult oral cancer patients? Is there any limit related with this effect?

Tobacco consumption in any form, smoking or chewing, has a close relationship with developing oral squamous cell carcinoma of the oral cavity (SCC). (Level of evidence 1+, Grade B Recommendation). The effect of excessive alcohol consumption is not as clear. (Level of evidence 2++, Grade B Recommendation). Concomitant consumption of tobacco and alcohol has a synergistic effect, increasing the likelihood of suffering from this type of oral cancer. (Level of evidence 1+, Grade B Recommendation).

Despite there being studies reporting a greater risk of developing SCC in patients starting the habits earlier, tobacco and alcohol do not appear to be the main risk factors in developing oral cancer in young patients, exposure time therefore possibly being a limiting factor of these habits. (Level of evidence 2++, Grade B Recommendation).

Patients who continue with the habit of smoking after diagnosis and/or treatment of oral cancer have a greater likelihood of experiencing a recurrence of the cancer or second primary malignant neoplasms. Additionally, patients who continue smoking and/or drinking have a larger number of complications in comparison with those who do not have these toxic habits. (Level of evidence 2+, Grade C Recommendation).

Clinical Question 2: In the preoperative period, when is the best time for undertaking the dental treatment?

The ideal time for undertaking pre-radiation therapy extractions is within the period between the second and fourth week prior to surgery, avoiding undertaking them within the 15 days prior to the start of radiation therapy (RT). (Level of evidence 2++, Grade B Recommendation). Undertaking them in the oncological pre-surgery period or simultaneously with it significantly reduces the delay in the start of radiation therapy treatment. (Level of evidence 2+, Grade C Recommendation).

Tartrectomy or subgingival curettage must be avoided in the 15 days prior to the start of RT, having to be carried out at an earlier time. (Level of evidence 2++, Grade B Recommendation).

Patients exposed to mouthwash with chlorhexidine (CHX) during and following the surgical and/or radiation therapy treatment have a higher risk of presenting osteoradionecrosis (ORN), therefore its use is not recommended in patients with oral cancer undergoing RT. (Level of evidence 2+, Grade B Recommendation).

The results do not allow it to be stated that there is a link between the endodontic treatment and a greater risk of ORN, although the authors who advocate it suggest avoiding undertaking it between two or four weeks prior to radiation therapy. (Level of evidence 2++, Grade B Recommendation).

Oral surgery, carried out in the pre-radiation therapy period, is associated with a significantly greater risk of developing ORN, therefore undertaking it must be avoided unless it is for oncological reasons. (Level of evidence 2++, Grade B Recommendation).

Topical application of fluoride gel and restorative treatment of cavities may be carried out at any time of the oncological pre-treatment, as they are not associated with a greater risk of ORN. (Level of evidence 2++, Grade B Recommendation).

Clinical Question 3: What actions in the pre-treatment period may reduce the incidence of mucositis in adult oral cancer patients?

The use of low frequency laser therapy in patients with oral cancer who will undergo radiation therapy or radiation therapy and chemotherapy simultaneously, is effective in the prevention of oral mucositis (OM). (Level of evidence 1++, Grade A Recommendation).

In patients who will undergo radiation therapy with doses lower than 50Gy, the use of mouthwashes with benzydamine is recommended (0.15%, 15ml every 3 or 6 hours, from the start of RT until two weeks after its completion) to prevent oral mucositis. (Level of evidence 1++, Grade A Recommendation).

The use of mouthwashes with benzydamine is suggested to prevent oral mucositis in patients who will undergo radiation therapy and chemotherapy or only chemotherapy. (Level of evidence 1+, Grade B Recommendation).

Food supplements have shown very limited evidence in improving the incidence of oral mucositis in patients who will undergo radiation therapy and chemotherapy. (Level of evidence 3, Grade D Recommendation).

The use of topical or systemic honey do not appear to clearly affect the incidence of mucositis or its grade, although it has demonstrated being effective in reducing the interruption of the oncological treatment and avoiding weight loss, without showing adverse effects. This means that its use can be suggested for patients who will undergo radiation therapy and/or chemotherapy, although its effect on other pathologies such as cavities must also be evaluated. (Level of evidence 1+, Grade B Recommendation).

Although there is not a high level of evidence, with regard to the effect on reduction of incidence of oral mucositis, it is recommended to carry out a dental check-up on patients who will undergo radiation therapy and/or chemotherapy, as well as providing the patient exhaustive instructions on oral hygiene. (Level of evidence 3, Grade D Recommendation).

The use of mouthwashes with saline and/or sodium bicarbonate is suggested, as they may help oral hygiene and improve discomfort caused by oral mucositis. (Level of evidence 4, Grade D Recommendation).

It is not recommended to use chlorhexidine mouthwashes for prevention of oral mucositis. (Level of evidence 1+, Grade B Recommendation).

During the design of the area to be radiated, it is recommended to minimise the impact of the oral mucosa, to avoid a greater effect and grade of oral mucositis. (Level of evidence 1+, Grade B Recommendation).

It is not possible to systematically recommend the use of intraoral devices to avoid the effect of radiation on the oral mucosa, although it is true that in some specific cases, due to tumour location, it may have a clinical benefit. (Level of evidence 3, Grade D Recommendation).

Clinical Question 4: What actions in the pre-treatment period may reduce the incidence of xerostomia and/or candidiasis in adult oral cancer patients?

There is sufficient evidence to recommend the use of intensity modulated radiation therapy for the prevention of acute and delayed xerostomia. (Level of evidence 1++, Grade A Recommendation).

The use of amifostine before radiation therapy sessions reduces the incidence of acute and delayed xerostomia. (Level of evidence 1++, Grade A Recommendation).

Acupuncture as a preventive treatment for xerostomia, in oral cancer patients who will receive head and neck radiation therapy requires greater evidence, and may be beneficial in the short-term, requiring more studies substantiating this data and evaluating the long-term effect. (Level of evidence 1+, Grade B Recommendation).

Submaxillary gland transfer surgery appears to be effective in the reduction of xerostomia associated with radiation therapy in selected patients, although more studies are needed to be able to implement it as a routine technique. (Level of evidence 1+, Grade B Recommendation).

There is no evidence of preventive treatments for candidiasis in cancer patients, although it is true that if facilitating factors of it are prevented, such as xerostomia or mucositis, a lower incidence would be expected. (Level of evidence 4, Grade D Recommendation).

Clinical Question 5: What actions in the preoperative period may reduce the incidence of cavities or periodontal disease in adult oral cancer patients?

Patients with a head and neck cancer are more likely to develop cavities and periodontal disease, although it is not exclusively related with the treatment to be carried out, the previous state of oral health having a noTable effect. (Level of evidence 2+, Grade D Recommendation).

Teaching and maintenance of oral hygiene is effective, as well as periodic check-ups with the dentist during the oncological treatment process. (Level of evidence 2+, Grade D Recommendation).

Although the precise time of finishing the preventive dental treatment is not specified, it is recommended to undertake extractions at least two weeks before beginning the oncological treatment. (Level of evidence 2+, Grade D Recommendation).

The authors also appear to agree on the importance of applying remineralising substances which may combat the side effects of xerostomia and hyposalivation which appear as a consequence of chemotherapy and/or radiation therapy. For this they recommend the application of fluoride and calcium phosphate, although they do not reach a consensus on the form, concentration or frequency of application. (Level of evidence 2+, Grade D Recommendation).

Clinical Question 6: Is there any standard for measuring quality of life in adult oral cancer patients? How is it related with therapeutic decisions?

There is no consensus on a questionnaire model in evaluating quality of life in adult head and neck cancer patients, although we did identify three tools which account for 90%. (Level of evidence 2++, Grade B Recommendation).

There is evidence that quality of life may be an important prognostic factor related with the clinical outcome obtained by the patient, and may therefore be susceptible to being included in hypothetical clinical decision making, at least in certain situations. (Level of evidence 2++, Grade B Recommendation).

There is no specific tool for evaluating quality of life related with health, which makes it difficult to integrate this idea in clinical practice. (Level of evidence 2+, Grade C Recommendation).

Clinical Question 7: What actions in the preoperative period may improve quality of life in adult oral cancer patients?

There is no clear indication that regulated dental treatment improves quality of life. (Level of evidence 1+, Grade B Recommendation). Measures intended to prevent tooth loss do have a direct effect on improving the quality of life of oral cancer patients. (Level of evidence 2+, Grade C Recommendation).

Clinical Question 8: What actions in the preoperative period may reduce the incidence of medication-related osteonecrosis or chemonecrosis in adult oral cancer patients?

Preventive and prophylactic dental measures in the preoperative period are able to reduce the incidence of medication-related osteonecrosis but do not prevent it completely in all cases. (Level of evidence 3, Grade D Recommendation).

Prior to beginning therapy with medications (oral or systemic bisphosphonates or denosumab) in adult oral cancer patients, they may undergo any dental treatment safely (endodontic, restorative, prosthetic, orthodontic, etc.) with the exception of surgical and periodontal treatments, which must be carried out a minimum of 4-8 weeks before the start of medication (Level of evidence 3, Grade D Recommendation).

Clinical 18/5/202318/5/2023Question 9: What actions in the preoperative period may reduce the incidence of osteoradionecrosis in adult oral cancer patients?

Prior to the oncological treatment, patients must be evaluated and treated for the oral pathology, due to entailing a greater risk of developing osteoradionecrosis of the maxillae. Endodontic, conservative, prosthetic and orthodontic treatments on teeth with a favourable prognosis must be carried out before the start of the therapy, while for teeth with pathologies or questionable or unfavourable prognosis, if the patient is subjected to an increased risk (high dose of RT > 55 Gy, mandibular molar, tooth close to the tumour, etc.) the teeth must be extracted. (Level of evidence 3, Grade D Recommendation).

The use of platelet rich plasma does not have beneficial impact on the incidence of this. (Level of evidence 3, Grade D Recommendation).

- Summary of recommendations during the oncological treatment

Clinical Question 10: What actions during the oncological treatment may reduce changes in taste in adult oral cancer patients?

It has not been possible to establish the absolute efficacy of any pharmacological strategy in the prevention of alterations of taste in oral cancer patients undergoing cancer treatment. (Level of evidence: 1++, Grade A Recommendation). Although zinc supplements have shown beneficial effects in non-cancer patients with idiopathic taste disorders or due to zinc deficiencies, its use is not recommended in patients with head and neck cancer undergoing radiation therapy, with or without chemotherapy. (Level of evidence: 1++, Grade A Recommendation).

The combination of pentoxifylline and vitamin E, as well as bethanechol, have revealed results which may be promising in the reduction of changes of taste in these patients. (Level of evidence: 1-, Grade D Recommendation).

It is recommended to refer patients to a nutritionist for dietary advice, especially in cases where they are suffering from malnutrition or at risk of suffering from it. (Level of evidence: 1-, Grade D Recommendation).

Clinical Question 11: What actions in the oncological treatment may reduce xerostomia and candidiasis in adult oral cancer patients?

There is evidence that supports the therapeutic role of pilocarpine, small doses (5mg/3/day) being the most suiTable for minimising adverse effects. (Level of evidence: 1+, Grade B Recommendation). Conversely, in terms of prevention there is not sufficient evidence to state the long-term beneficial effect of a specific drug, although the evidence is promising for amifostine or the C/E vitamin complex. (Level of evidence: 1+, Grade B Recommendation).

With regard to candidiasis, there is strong evidence supporting the efficacy of drugs absorbed (fully or partially) in the digestive tract in prevention of the case. (Level of evidence: 1++, Grade A: Recommendation). As a systemic agent, fluconazole is a good option due to the demonstrated benefit, as well as its safety. For topical treatment, both miconazole and clotrimazole may be good options. (Level of evidence: 1+, Grade B Recommendation).

There is no conclusive evidence that drugs absorbed fully in the digestive tract (fluconazole, ketaconazole and itraconazole) are effective for reducing the incidence of candidiasis in oral cancer patients. (Level of evidence: 1+, Grade B Recommendation).

Clinical Question 12: What actions in the oncological treatment may reduce mucositis in adult oral cancer patients?

All measures adopted for the treatment of oral mucositis resulting from the oncological treatment, mouthwash with benzydamine, low frequency laser with or without oral care protocol, and mouthwash with morphine at 2% for pain reduce the incidence of OM in patients treated for oral cancer. Although oral care is essential for oral mucositis induced by chemoradiation therapy, it cannot prevent serious oral mucositis in itself. Even so, it contributes to the previous treatments mentioned. (Level of evidence: 1+, Grade A Recommendation).

Photobiomodulation with low frequency laser reduces the incidence and severity of mucositis in patients treated with radiation therapy and/or chemotherapy. (Level of evidence: 1+, Grade A Recommendation).

Benzydamine hydrochloride applied as mouthwash acts as an anti-inflammatory agent, significantly reducing both erythema and ulceration. Benzydamine significantly reduces OM even in doses > 50 Gy in patients with oral cancer. Its function in patients who receive concomitant chemotherapy must be more thoroughly evaluated. (Level of evidence: 1-, Grade B Recommendation).

Clinical Question 13: In what situations is it justified to carry out bloody dental treatments during the oncological treatment period?

Extraction in the lower maxilla within the radiation field in patients with a dose of radiation greater than 60 Gy represents a greater risk of developing ORN. If the extraction is carried out post-radiation, a series of prophylactic measures must be taken between operations, such as alveoloplasty, primary closure and limited periosteal trauma during the extraction. In turn, the number of teeth to be extracted in one session must be limited, and low-adrenaline local anaesthetic must be used. (Level of evidence: 2++, Grade A Recommendation).

Patients treated with doses lower than 50 Gy have a risk of losing the implant similar to that of non-irradiated patients. Therefore, exposure from 50 to 65 Gy must not be considered a limit for treatment with implants. (Level of evidence: 2+, Grade B Recommendation).

Periodontal surgery may be carried out on patients after radiation therapy, although future research is necessary in this field. (Level of evidence: 2-, Grade B Recommendation).

Medication with bisphosphonates may affect orthodontic movements, affecting the osteoblastic activity and above all osteoclasts. Even so, a dental extraction in an irradiated patient medicated with bisphosphonates is possible, avoiding an extraction and greater risk of osteoradionecrosis. (Level of evidence: 3, Grade D Recommendation).

Clinical Question 14: What actions in the oncological treatment may reduce the appearance of cavities and periodontal lesions in adult oral cancer patients?

There is a greater predisposition of suffering cavities, periodontal disease, xerostomia, mucositis or fungal infections such as candidiasis and it is recommended to carry out periodic dental check-ups. During these appointments, x-rays must be carried out, which help to diagnose the incipient disease, in additional to clinical examinations and application of topical fluoride. (Level of evidence 2+, Grade D Recommendation).

- Summary of recommendations after the oncological treatment

Clinical Question 15: What data is necessary in an oncological discharge report to ensure good postoperative dental treatment?

The Survival Care Plan must include diagnosis of the primary cancer TNM and treatment. With regard to treatment, it must include:

1. Surgical: type of surgical approach (conventional, robotic surgery, transoral laser microsurgery) and type of reconstructive surgery (in case of having been carried out).

2. Chemoradiation therapy:

a. Type of fractioning of the radiotherapy: Altered fractioning, hypofractioning or normofractioning. In this way the likelihood of acute or delayed toxicities can be ascertained.

b. Radiation dose: quantity of radiation that the patient has received, as well as the duration of the treatment, the time that has passed since the treatment ended, whether or not it has been combined with chemotherapy and radiation zone.

i. Concomitant radiation therapy boost

ii. Intensity modulated radiation therapy

c. Other: volumetric modulated arc, stereotactic radiation therapy, hypofractionated stereotactic body radiation therapy

3. Hematopoietic cell transplant therapy

4. Medical oncology: treatment and management of symptoms of acute or chronic oral complications. (Level of evidence 3, Grade D Recommendation).

Clinical Question 16: What oncological post-treatment periods are appropriate for undertaking different dental actions?

Extractions are not recommended for 2 to 5 years post-RT, and especially in the first 3 months post-RT. (Level of evidence 2+, Grade B Recommendation). Other studies conclude that dental extractions carried out in a period of 6 months or fewer post-RT reduce the risk of ORN compared with those carried out later on (Level of evidence 2+, Grade B Recommendation). Effort must be made to not subject the patient to extractions before and after RT, as the risk of ORN is even greater. (Level of evidence 2-, Grade C Recommendation).

There is a clear relationship between the risk of ORN and the total dose of the treatment with RT. Doses greater than 50-60 Gy are decisive in the increase of the risk. This factor must be taken into account, requesting a report from those responsible for oncological treatment. Systemic illnesses such as diabetes and chemotherapy associated with RT increase the risk of ORN. (Level of evidence 2-, Grade C Recommendation).

The number of extractions carried out in a single session being >5 increases the risk of ORN. As a result, it is important to limit the number of total extractions as well as the number of simultaneous extractions. (Level of evidence 2+, Grade B Recommendation).

Oral surgery in the 6 months following RT has a high risk and increases if combined with chemotherapy. (Level of evidence 2+, Grade B Recommendation).

Endodontics with or without coronectomy to access the instrumentation of the channels or to avoid an extraction post-RT may reduce the risk of ORN (Level of evidence 4, Grade D Recommendation). The level of success is high if carried out before the RT. (Level of evidence 3, Grade D Recommendation).

Fillings of cavities do not represent any increased risk related with ORN (Level of evidence 4, Grade D Recommendation). The prognosis of fillings reduces based on the number of faces of a tooth filled. (Level of evidence 3, Grade D Recommendation).

Removable prostheses must be avoided in the first year post-RT (Level of evidence: 4. Grade D Recommendation). There is evidence in terms of reduction of complications, above all in the first six months post-RT. (Level of evidence 2+, Grade C Recommendation).

Fixed prostheses increase the risk of accumulation of plaque on the coronary margin, associated with hygiene difficulties and xerostomia, therefore being advised against. In the case of being suggested, the marginal preparations must be supragingival to facilitate hygiene and control of cervical cavities. (Level of evidence 4, Grade D Recommendation).

Supra and subgingival curettage post-RT is a risk factor for ORN. (Level of evidence 4, Grade D Recommendation).

Clinical Question 17: In what situations is undertaking palliative dental actions justified?

With regard to conservative palliative dental treatments, there is no scientific evidence with regard to their efficacy, prognosis or different techniques, comparing their results, although it must be taken into account that their design may not be ethical. (Level of evidence 4, Grade D Recommendation).

Oral hygiene care and disinfection of prostheses are absolutely necessary in palliative care patients as they improve their quality of life and contribute to maintaining nutrition and hydration. This care must be carried out by the patient themselves if they are capable of doing so, or by their carers, whether relatives or healthcare personnel. (Level of evidence 2-, 4, 4, 4, 2++, Grade C Recommendation).

Management of the treatment of mucositis, xerostomia and dry mouth is widely tested. Palliative treatments must be carried out aimed at improving this condition given its high prevalence. (Level of evidence 2-, 4, 2-, 2+, 4, 4, 4, Grade C Recommendation).

In the treatment of oral candidiasis in terminal patients, a single 150mg oral dose of fluconazole has shown to be effective, despite the fact that if they do not have a good therapeutic response or show resistance, the treatment must be prolonged, or it will be necessary to carry out a resistance study. (Level of evidence 2-, Grade C Recommendation).

Complementary palliative treatment through acupuncture or with electroacupuncture may be effective in reducing pain and increasing saliva flow and does not show adverse reactions, although studies that support these therapies are very heterogeneous. (Level of evidence 2++, Grade C Recommendation).

Clinical Question 18: After the oncological treatment, what is the treatment of choice for osteoradionecrosis based on its stage?

There is not a standardised protocol for the treatment of ORN, although it must be multimodal and must be adapted to the stage and comorbidity of the patient, conservative therapy always having to be chosen as a first option. Resective surgery and mandibular reconstruction should be reserved for the most severe cases. (Level of evidence: 1+, Grade B Recommendation).

In the initial stages (corresponding to stage I of the classifications by Marx, Epstein, Notani and Lyons) conservative treatment based on taking antibiotics (in case of infection), analgesics, strict oral hygiene, saline irrigation, use of hyperbaric oxygen combined with debridement and removal of the affected teeth are recommended. (Level of evidence: 3, Grade D Recommendation).

In the intermediate stages (corresponding to stage II of the classifications by Marx, Epstein, Notani and Lyons) two pharmacological regimens are recommended: the first with antibiotics, anti-inflammatories and antifungals as prior therapy, subsequently taking pentoxifylline, tocopherol and clodronate, combined with undertaking sequestrum or alveolar mandibulectomy when necessary, as this would accelerate healing (Level of evidence: 3, Grade D Recommendation).

In the advanced stages (corresponding to stage III of the classifications by Marx, Epstein and Notani and stages III and IV by Lyons) and aggressive or recurrent cases (in which conservative and pharmacological treatment of the previous stages has not worked), mandibular resective treatment and reconstruction with bone graft are recommended, grafts free of fibula being those which show a higher success rate. (Level of evidence: 1+, Grade B Recommendation).

Clinical Question 19: After the oncological treatment, what is the treatment of choice for osteochemonecrosis or medication-related osteonecrosis based on its stage?

Additionally, in the case of osteochemonecrosis, it has been observed that the surgical treatment will provide a more rapid response and with a greater likelihood of healing than the non-surgical conservative treatment.

In patients at risk, only patient education on this issue should be incorporated; no treatment is necessary. In stage 0 it is recommended for the treatment to be systemic and based on pain control (analgesics) and on the appearance of potential infections (antibiotics). It is important to monitor the patient with periodic controls and x-rays (appearance of sclerosis or extractions which are slow to heal). It is also recommended for elderly patients, patients who are not candidates for surgery or cancer patients with palliative treatment. (Level of evidence: 2+, Grade D Recommendation).

In stages 1 and 2 conservative surgical treatment is recommended based on debridement with elimination of potential sequestrum and in combination with contributing conservative treatment: 0.12% chlorhexidine mouthwash, microbial cultivation to guide the antibiotic regime (in case of associated infection, presence of fistula) with penicillin or quinolones, metronidazole or clindamycin in case of allergies and analgesic regime. Another recommended treatment is hyperbaric oxygen. (Level of evidence: 2+, Grade D Recommendation).

In stage 3 surgical treatment is recommended combined with antibiotic and analgesic treatment. The surgical treatment will vary depending on the size of the lesion: surgical debridement, alveolectomy, marginal or segmental mandibulectomy. These latter two treatments are recommended for the most aggressive cases and may be accompanied by reconstructive surgeries (microvascularised free graft). (Level of evidence: 1-, Grade D Recommendation).

Clinical Question 20: After the oncological treatment, what is the treatment of choice for xerostomia based on its stage?

The indications and results with regard to use of pilocarpine and cevimeline for treatment of xerostomia caused by radiation therapy cannot be related based on the grade of glandular impact prior to their administration. Their effect is determined by the grade of glandular preservation. The adverse effects of pilocarpine may be a restriction on its suggestion. The data found on its efficacy is based on comparisons before and after its use with regard to the increase of saliva flow or a VAS scale, or compared to placebo, without determining these effects based on the grade of xerostomia (Level of evidence: 1+, 2++, 2++, 4. Grade B Recommendation). The grade of recommendation cannot be established with regard to the treatment based on the grade of severity of xerostomia.

Amifostine demonstrated its efficacy for reducing the risk of developing grade 3-4 mucositis, grade 2-4 acute xerostomia, or delayed grade 2-4 (40% reduction of risk) or grade 3-4 of dysphagia, but was not effective in the subgroup of patients undergoing concomitant treatment with chemotherapy. Although it may be debaTable, it does not have a tumour protective effect, but presents adverse effects. (Level of evidence: 1++, Grade A Recommendation).

Depending on the grade of impact, different actions are proposed: grade 1, no action; grade 2, saliva substitutes, sugar-free sweets or chewing gum and sialogogues occasionally; grade 3, the same measures used with greater frequency; and grade 4, the use of saliva substitutes, water in food, sugar-free sweets, chewing gum and sialogogues. (Level of evidence: 4, Grade of Recommendation: A grade of recommendation cannot be established).

Malic acid or sour sweets with added calcium are effective and reduce the risk of the appearance of cavities due to dissolving of the hydroxyapatite. (Level of evidence: 2-, Grade of Recommendation: A grade of recommendation cannot be established).

Bethanechol reduced the severe grade (80.5%, 75.7%, 70%) and increased the proportion of medium grade (at 1, 2 and 3 months: 19.5%, 24.3%, 30%). There were no differences in the saliva flow between the same times. (Level of evidence: 2+, Grade C Recommendation).

Phytotherapy, thymol and honey are effective in the treatment of xerostomia. Thymol mouthwash with honey at 7 weeks reduced xerostomia in grade 3 and 4 patients by 25% (*p*<0.003). (Level of evidence: 2+, 1+, Grade B Recommendation). Visco-Ease® has not demonstrated its efficacy. (The level of evidence cannot be established).

Hyperbaric oxygen has beneficial effects with regard to xerostomia related with the grades of impact. Only one study measured the value of VAS (8 before the OHB sessions and 5 afterwards in patients with saliva flow <0.1ml/min, 4.5 before and 3 afterwards when the saliva flow was 0.1ml/min). Results were not found linked with the grade of severity of xerostomia and therapy with OHB in the rest of the studies included. (Level of evidence: 2+, 1+, 3, Grade B Recommendation).

The studies that evaluate the efficacy of TENS report significant differences with regard to the flow increase after application, but not those related with the grade of severity before and after the xerostomia (Level of evidence: 3, Grade D Recommendation. The grade of recommendation cannot be established with regard to the treatment based on the grade of severity of xerostomia.

Invasive treatments such as submaxillary gland reimplant or infiltration guided by mesenchymal stem cell ultrasound, although offering good results, refer to studies with a limited number of patients or with short monitoring and do not compare their results based on the grade of intensity of the xerostomia. (Level of evidence: 1+. 3, Grade B Recommendation). A grade of recommendation cannot be established for the glandular reimplantation. The grade of recommendation cannot be established with regard to the treatment based on the grade of severity of xerostomia.

Clinical Question 21: What are the suggestions and periods for prosthetic and implantology rehabilitation of adult oral cancer patients?

Prosthetic rehabilitation, whether conventional or implant-supported, of patients treated for oral cancer significantly improves their quality of life and masticatory function. (Level of evidence: 2, Grade B Recommendation).

Implant-supported prostheses provide the patient better functional results than conventional prostheses, without the differences being significant with regard to quality of life or satisfaction of the patient, therefore their suggestion must be for cases in which an evident functional deficiency with conventional prostheses is confirmed or predicted. (Level of evidence: 2, Grade C Recommendation).

The studies analysed do not allow the suggestion that simultaneous or deferred fitting of implants affect their survival once they are subjected to loads. Although the simultaneous technique entailed a significant reduction of time until the fitting of the final prosthesis, it entailed a high percentage of implants that did not end up being loaded. (Level of evidence: 2, Grade C Recommendation).

A 12-14-month interval of time is considered necessary from completion of the oncological treatment until the fitting of implants. (Level of evidence: 2, Grade D Recommendation). Periods of osseointegration shorter than 6 months are significantly associated with the loss of implants. (Level of evidence: 2, Grade C Recommendation). There are no differences in survival between implants fitted in the maxilla or mandible, but more failures are observed in the rear area [120, 121]. (Level of evidence: 3, Grade D Recommendation).

Treatment with RT and the nature of the bone tissue negatively affect the survival of the implants, showing worse results for irradiated patients and for implants fitted in grafted bone. (Level of evidence: 2, Grade B Recommendation).

The prevalence of periimplantitis in these patients differs depending on the authors, although they agree in indicating that the local and general conditions in these patients make it more likely than in the general population. (Level of evidence: 3, Grade D Recommendation). Patients treated for oral cancer present a markedly greater incidence of peri-implant hyperplastic lesions than the general population, associated with the absence of keratinised mucosa inserted, given its more frequent occurrence in alveolar mucosa and in skin grafts. (Level of evidence: 2, Grade C Recommendation).

The little data reported on the incidence of ORN in these patients reveals that it is not the main cause of implant failure, although it may be its origin. Smokers have a higher risk of presenting ORN, an active tobacco habit constituting a predictive risk factor for its development. (Level of evidence: 2, Grade C Recommendation).

## Discussion

The incorporation of the Figure of the dentist in hospitals and cancer patient care centres is considered essential.

Due to the demographic growth of the elderly population and of patients with oral cancer, it would be recommended to incorporate additional content into undergraduate and post-graduate subjects, teaching students about early diagnosis of oral cancer, as well as dental care for cancer patients.

The main limitation when analysing the results obtained is the wide diversity of treatment protocols for each clinical question raised. This caused substantial difficulties in being able to obtain more robust results.

Another significant aspect to take into account is the absence of replication of results, which would lead to greater evidence available on which to base the recommendations.

As a result of the extensive bibliography consulted to prepare this clinical practice guideline, there is a need to carry out a larger number of randomised controlled clinical trials, with a sufficiently representative sample size and using internationally accepted and validated tools.
